# Fanconi anemia signaling and Mus81 cooperate to safeguard development and crosslink repair

**DOI:** 10.1093/nar/gku676

**Published:** 2014-07-23

**Authors:** Meghan Larin, David Gallo, Laura Tamblyn, Jay Yang, Hudson Liao, Nestor Sabat, Grant W. Brown, J. Peter McPherson

**Affiliations:** 1 Department of Pharmacology and Toxicology, University of Toronto, Toronto, M5S 1A8, Canada; 2 Department of Biochemistry, Terrence Donnelly Center for Cellular and Biomolecular Research, University of Toronto, Toronto, M5S 3E1, Canada

## Abstract

Individuals with Fanconi anemia (FA) are susceptible to bone marrow failure, congenital abnormalities, cancer predisposition and exhibit defective DNA crosslink repair. The relationship of this repair defect to disease traits remains unclear, given that crosslink sensitivity is recapitulated in FA mouse models without most of the other disease-related features. Mice deficient in Mus81 are also defective in crosslink repair, yet *MUS81* mutations have not been linked to FA. Using mice deficient in both Mus81 and the FA pathway protein FancC, we show both proteins cooperate in parallel pathways, as concomitant loss of FancC and Mus81 triggered cell-type-specific proliferation arrest, apoptosis and DNA damage accumulation *in utero*. Mice deficient in both FancC and Mus81 that survived to birth exhibited growth defects and an increased incidence of congenital abnormalities. This cooperativity of FancC and Mus81 in developmental outcome was also mirrored in response to crosslink damage and chromosomal integrity. Thus, our findings reveal that both pathways safeguard against DNA damage from exceeding a critical threshold that triggers proliferation arrest and apoptosis, leading to compromised *in utero* development.

## INTRODUCTION

Fanconi anemia (FA) is an inherited disease with afflicted individuals susceptible to bone marrow failure, congenital anomalies and/or cancer (1,2). FA is linked to mutations in one of the 16 known FANC genes, which encode components of a common molecular pathway that respond to interstrand crosslink (ICL) damage and other lesions that compromise DNA replication (2,3). Damage triggered by ATR activation via FANCM/FAAP24 and FANCJ/TopBP1 complexes serves to prepare FANCD2 and FANCI for monoubiquitination by the FA core complex (FANCA, B, C, E, F, G, L and M, together with the FA-associated proteins FAAP24, FAAP100, MHF1 and MHF2). Monoubiquitinated FANCD2-FANCI is recruited to DNA damage sites in chromatin, where they facilitate the activation of downstream repair events that utilize translesion synthesis, lesion removal and homologous recombination to restore DNA integrity. This series of events likely includes stepwise conversion of DNA lesions and repair intermediates through the coordinated action of structure-specific endonucleases that may include FAN1, SNM1A, XPF-ERCC1, SLX1-SLX4 and MUS81-EME1 (4–9). The latter two nucleases have also recently been shown to choreograph cleavage events that resolve Holliday junctions (HJs), a requisite step in homologous recombination. Although the majority of mitotic crossovers that occur in mammalian cells are generated by the coordinated action of MUS81-EME1 and SLX4-SLX1 (10–12), it remains to be clarified whether these nucleases participate in crosslink repair via HJ resolution or action on distinct repair intermediates (13). Recent studies have demonstrated that mutations in *SLX4* (7,13) and *ERCC4/XPF* (14) can result in FA (FA-P and -Q, respectively); however, potential links between MUS81-EME1 and human FA have not been demonstrated.

Although tremendous strides have been made in our understanding of molecular events that lead to crosslink recognition and repair by FA proteins, the underlying mechanisms linking disease-associated repair defects to pathology remain largely unknown. One reason for this is that mouse models of FA largely fail to recapitulate many of the prevalent features of the human disease (15,16). In particular, all mouse models of FA display ICL sensitivity but show varying degrees of overlap with other attributes, which calls into question whether loss of ICL repair capacity alone is sufficient for triggering disease traits or if other factors are involved. Establishing whether FA is due to the specific ICL sensitivity is complicated by additional roles for FA proteins in DNA transactions outside of ICL repair, such as homology-directed repair of double-strand breaks and nucleotide excision repair (3), in addition to numerous interactions of these proteins with other pathways unrelated to DNA repair (17–23).

The structure-specific endonuclease MUS81-EME1 participates in ICL repair, yet the exact role of this nuclease in the processing of these lesions remains unclear. Several models of replication-dependent ICL repair propose that MUS81-EME1 acts together with XPF-ERCC1 to create incisions flanking the damaged region of DNA to be repaired (3,9,13,24–26). In addition, the cleavage activity of MUS81-EME1 may also serve to convert replication fork structures to a repair intermediate that generates a double-strand break (27). Whether or not MUS81-EME1 participates in the FA pathway or another separate pathway of ICL repair is unclear, although a recent study indicates that MUS81-EME1 nuclease activity is stimulated by interaction with FANCA (28).

FA signaling and Mus81 have both been linked to common DNA repair pathways that respond to DNA crosslinks and replication-associated DNA damage, yet there are marked differences when either FA signaling or Mus81 is disrupted *in vivo*. *FancC^−/−^* mice exhibit ICL sensitivity, partial to complete sterility, microphthalmia and susceptibility to *in utero* lethality; however, other human FA-associated traits are mild or absent (29,30). In contrast, although mice deficient in Mus81 also exhibit ICL sensitivity, they appear phenotypically normal, are born at normal Mendelian ratios and exhibit a propensity for lymphoma development in a mixed strain background (31) but not when backcrossed into a BL/6 background (Larin,M. and McPherson,J.P., unpublished observations).

Although SLX4, ERCC4/XPF and MUS81 reside in a structure-specific nuclease complex and mutations in SLX4 and ERCC4/XPF can result in FA, a possible link between MUS81 and FA remains to be established. Here, we have crossed *FancC^+/−^ (F^het^)* mice to mice deficient in Mus81 activity (*Mus81^−/−^ or M^ko^*) to generate *F^ko^M^ko^* mice. We find that FA and Mus81 cooperate to ensure genome integrity during development. Concomitant loss of FA and Mus81 exacerbates ICL sensitivity with a corresponding increase in developmental defects and impaired growth that more closely resemble human FA disease traits. Our findings suggest that loss of ICL repair capacity alone is sufficient for triggering these traits.

## MATERIALS AND METHODS

### Mice

*FancC^−/−^* (*F^ko^*) mice (29) and *Mus81^−/−^* (*M^ko^*) mice (31) were maintained on a C57BL/6 background. To minimize impact of reduced fertility in *FancC^−/−^* mice, *FancC^−/−^ - Mus81^−/−^* (*F^ko^M^ko^*) mice were obtained through crosses between *FancC^+/−^ - Mus81^−/−^* x *FancC^+/−^ - Mus81^+/−^* or *FancC^+/−^ - Mus81^−/−^* x *FancC^+/−^ - Mus81^−/−^* crosses. X-ray imaging was performed using a Faxitron Cabinet Digital Radiography System (Faxitron BioOptics, Tuscon, AZ, USA). Images were taken using a voltage of 24 kV for 15 s. Blood collected by saphenous vein bleeds was analyzed for Complete Blood Counts at the Toronto Centre for Phenogenomics, Mount Sinai Hospital, Toronto, Canada using a Hemavet Hematology Analyzer (950FS). Incidence of micronuclei (Howell-Jolly bodies) was quantitated from tail blood smears from 6-month old mice stained with Accustain® Wright-Giemsa Stain, Modified (Sigma-Aldrich). All experiments were performed in compliance with the Ontario Cancer Institute animal care committee guidelines.

### Embryo analysis

Pregnant mice were injected with bromodeoxyuridine (BrdU) (0.1-mg BrdU/g mouse weight) using intraperitoneal injection 45 min prior to sacrifice and embryo collection. Harvested embryos were deemed to be alive by the observation of cardiac contractions. Mouse embryos were fixed, dehydrated and processed according to standard protocols. Paraffin-embedded embryos were sectioned and used for TUNEL analysis or immunostained for BrdU or γH2AX. For TUNEL analysis, sections were incubated at 37°C for 15 min with proteinase-K (50 mg/ml in 10-mM Tris, pH 7.5) and then labeled according to the protocols provided in the *in situ* cell detection kit, fluorescein (Roche, Germany). BrdU immunostaining was conducted essentially as described previously (31) using anti-BrdU antibody raised in rat (Abcam, ab6326) in Histoblock blocking buffer, followed by goat anti-rat Alexa Fluor® 568 secondary antibody (Invitrogen, A11077). For γH2AX immunostaining, sections were incubated with anti-γH2AX antibody raised in mouse (Abcam, ab2893-50) after antigen retrieval, followed with goat anti-mouse Alexa Fluor® 568 secondary antibody. For all immunofluorescence experiments, slides were mounted using Vectashield mounting media with 4′,6-Diamidino-2-Phenylindole (DAPI). Sections were imaged using an AxioImager.ZI epifluorescence microscope, AxioCamHR camera and Axiovision software (Zeiss). In BrdU-stained sections, the exposure times for each set of litter siblings were determined using the most brightly stained section in the set. BrdU images were quantified for total cell number and overall intensity using images from the branchial arch (×20 magnification) using Metamorph software (Olympus). The number of positive γH2AX and TUNEL cells was assessed in the forebrain region of each embryo and quantified based on the surface area of the neural epithelium of each forebrain region. Surface area was calculated using Axiovision software (Zeiss).

### Embryonic fibroblasts

Immortalization of embryonic fibroblasts with Simian virus large T antigen, clonogenic assays, cell-cycle analysis, γH2AX staining by flow cytometry and metaphase analysis were performed as previously described (32). For micronuclei enumeration, at least 200 cells/genotype were scored in triplicate. For metaphase analysis, between 15 and 50 metaphases were scored/genotype. For proliferation assays, passage 1 (p1) primary or immortalized fibroblasts were seeded at 5 × 10^5^ per well/dish in triplicate. Cells were counted and re-seeded at the starting density every 2 or 3 days for primary or immortalized cells, respectively. Apoptosis was quantified using an FITC Annexin-V Apoptosis Detection Kit I (556547, BD Bioscience) and flow cytometry using an FACS Calibur with Cell Quest software (Becton Dickenson) and analysis performed using FloJo software.

### Replication fork velocity

Primary fibroblasts were exposed to 50-nM mitomycin-C for 24 h and allowed to recover in drug-free media for 6 h, with CldU and IdU incorporation occurring in successive 30-min intervals before harvest. CIdU and IdU staining of labeled DNA fibers from primary murine embryonic fibroblasts (MEFs) was carried out as described (33) with the following modifications in the plug washing and melting steps: following proteinase K digestions plugs were washed 5 × 10 min in 10-ml TE_50_ buffer (10-mM Tris-HCL pH 7.0, 50-mM ethylenediaminetetraacetic acid). One plug was transferred to a round bottom polycarbonate tube with 100-μl 6.7-μM YOYO-1 (Y3601; Invitrogen) in TE_50_ for 30 min at room temperature in the dark to stain genomic DNA. Plugs were washed 3 × 5 min in 10-ml TE_50_ and incubated in for 5 min in 5 ml of 50-mM MES buffer at pH 5.7. The MES buffer was replaced with a fresh 2 ml and heated to 72°C for 15–20 min to melt the agarose plugs. Following staining and coverslip mounting, images were taken of between 100 and 150 fibers per sample at ×63 magnification using an Imager.ZI fluorescence microscope and Axiovision software (Zeiss). Replication fork velocities from at least two independent experiments were pooled for the final distribution.

### Statistical analysis

Parametric data were analyzed by one or two-way analysis of variance (ANOVA; Holm-Sidak or Bonferroni post hoc analysis). Non-parametric data were assessed by one or two-way ANOVA on ranks, followed by post hoc analysis for significant results. Data analysis was completed using SigmaPlot 11.0 software. Replication fork rates were analyzed by the Mann–Whitney U test using R statistical software. A factor was considered statistically significant if a two-sided *P* value <0.05.

## RESULTS

### FancC and Mus81 cooperate to ensure normal development

**Figure 1.**
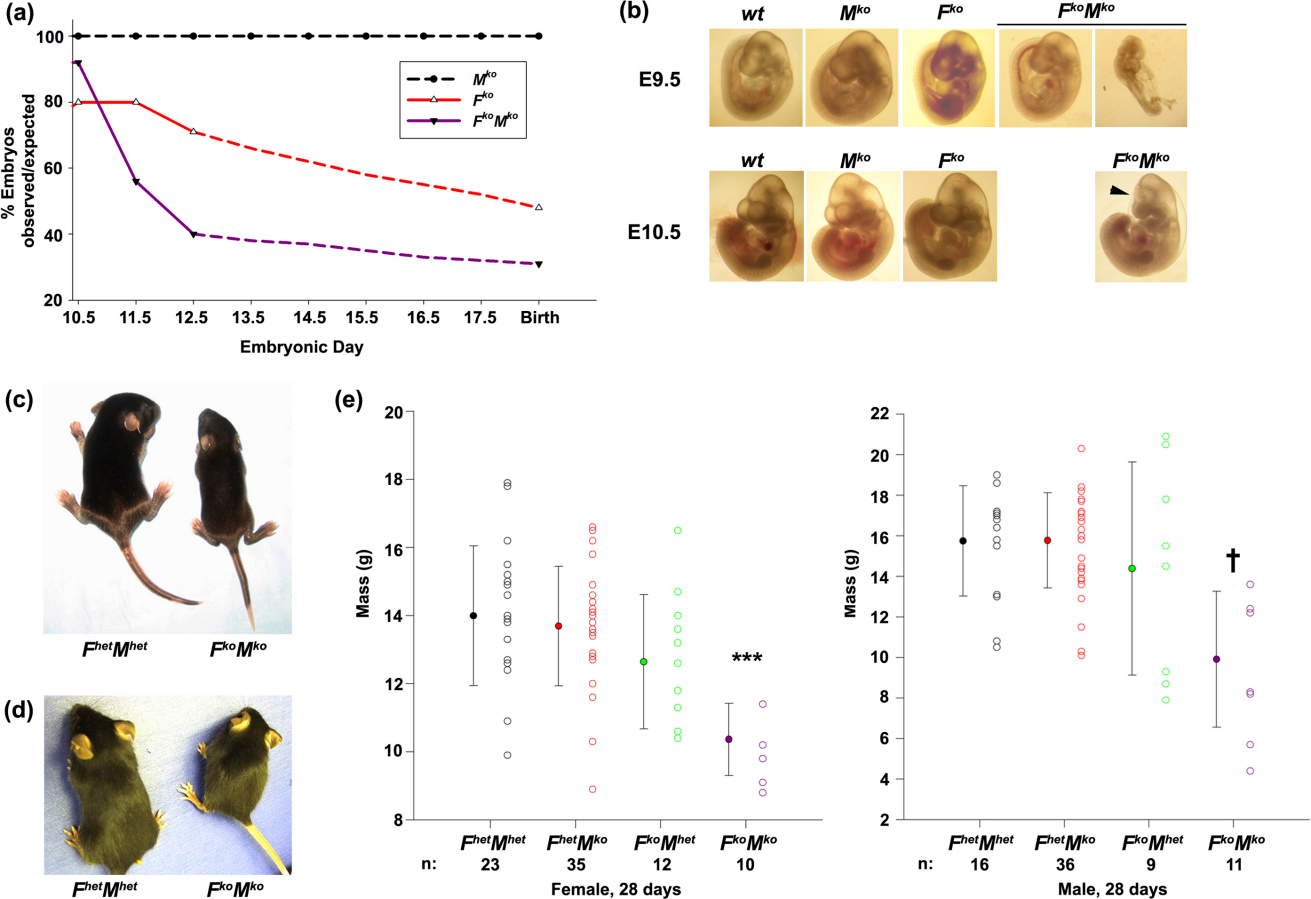
Increased embryonic lethality and growth delay in *F^ko^M^ko^* mice. (**a**) Percentage of observed/expected *F^ko^M^ko^*, *F^ko^* and *M^ko^* embryos/embryonic day (solid line) and projected survival (dashed line). (**b**) Representative images of developmental delays in *F^ko^M^ko^* embryos at E9.5 and E10.5. Arrowhead, microcephaly. (**c**) Growth delay in *F^ko^M^ko^* mice compared to sibling *F^het^M^het^* mice at 2 weeks of age. (**d**) Growth delay in *F^ko^M^ko^* mice compared to sibling *F^het^M^het^* mice at 6 weeks of age. (**e**) Mass of female and male *F^het^M^het^, F^het^M^ko^, F^ko^M^het^* and *F^ko^M^ko^* mice at 4 weeks of age. Open circles represent each mouse assessed, solid circles represent mean mass for each group. Error bars represent ± standard deviation (SD), *n* values on the x-axis denote sample sizes. For the female cohort, ***P < 0.001 for *F^ko^M^ko^* versus all genotypes by one-way ANOVA followed by Holm–Sidak post hoc test. For the male cohort, †*P* < 0.05 *F^ko^M^ko^* versus *F^het^M^het^* or *F^het^M^ko^* by one-way ANOVA on ranks followed by Dunn's post hoc test.

To query a possible interaction between the FA pathway and Mus81 *in vivo*, *F^het^M^ko^* mice or *F^het^M^het^* mice were intercrossed to obtain mice deficient in both FancC and Mus81. Of the 270 offspring resulting from *F^het^M^ko^* × *F^het^M^het^* crosses, 26 *F^ko^M^ko^* mice were expected but only nine were observed at birth, indicating significant *in utero* lethality (*P* < 0.005, χ^2^ test; Supplementary Table S1). Of the 245 offspring obtained from *F^het^M^ko^* × *F^het^M^ko^* crosses, 61 *F^ko^M^ko^* mice were expected but only 25 *F^ko^M^ko^* mice were obtained at birth, also indicative of embryonic lethality (*P* < 0.005, χ^2^ test). The percentage of observed/expected *F^ko^M^ko^* mice at birth for both crosses was 35% and 41%, respectively, indicative of a greater susceptibility to death *in utero* than *F^ko^* mice (61% observed/expected; Supplementary Table SI). Recovery of viable embryos from E9.5 and earlier revealed that *F^ko^M^ko^* embryos were present and viable at the expected Mendelian ratio, but a drastic decline in viable *F^ko^M^ko^* embryos occurred between E10.5 and E12.5 (from 79% observed/expected at E9.5 to 40% by E12.5) compared to *F^ko^* embryos (from 67% observed/expected at E9.5 to 62.5% by E12.5; Figure 1A and Supplementary Table S1). Unlike *F^ko^* embryos, *F^ko^M^ko^* embryos appear particularly susceptible to death during this developmental period. When we examined littermates between E9.5 and E11.5, viable *F^ko^M^ko^* embryos exhibited a greater incidence of delays in growth and development, an effect particularly prevalent in the head region (Figure 1B and Supplementary Table S2). The growth defect observed in *F^ko^M^ko^* embryos was also evident in live-born mice (Figure 1C–E). Mass of *F^ko^M^ko^* mice and sibling controls was measured on days 10, 21, 28, 42 and 56. Both male and female *F^ko^M^ko^* mice at day 28 were smaller in size and mass than *F^ko^*, *M^ko^* or littermate controls (Figure 1E).

**Figure 2.**
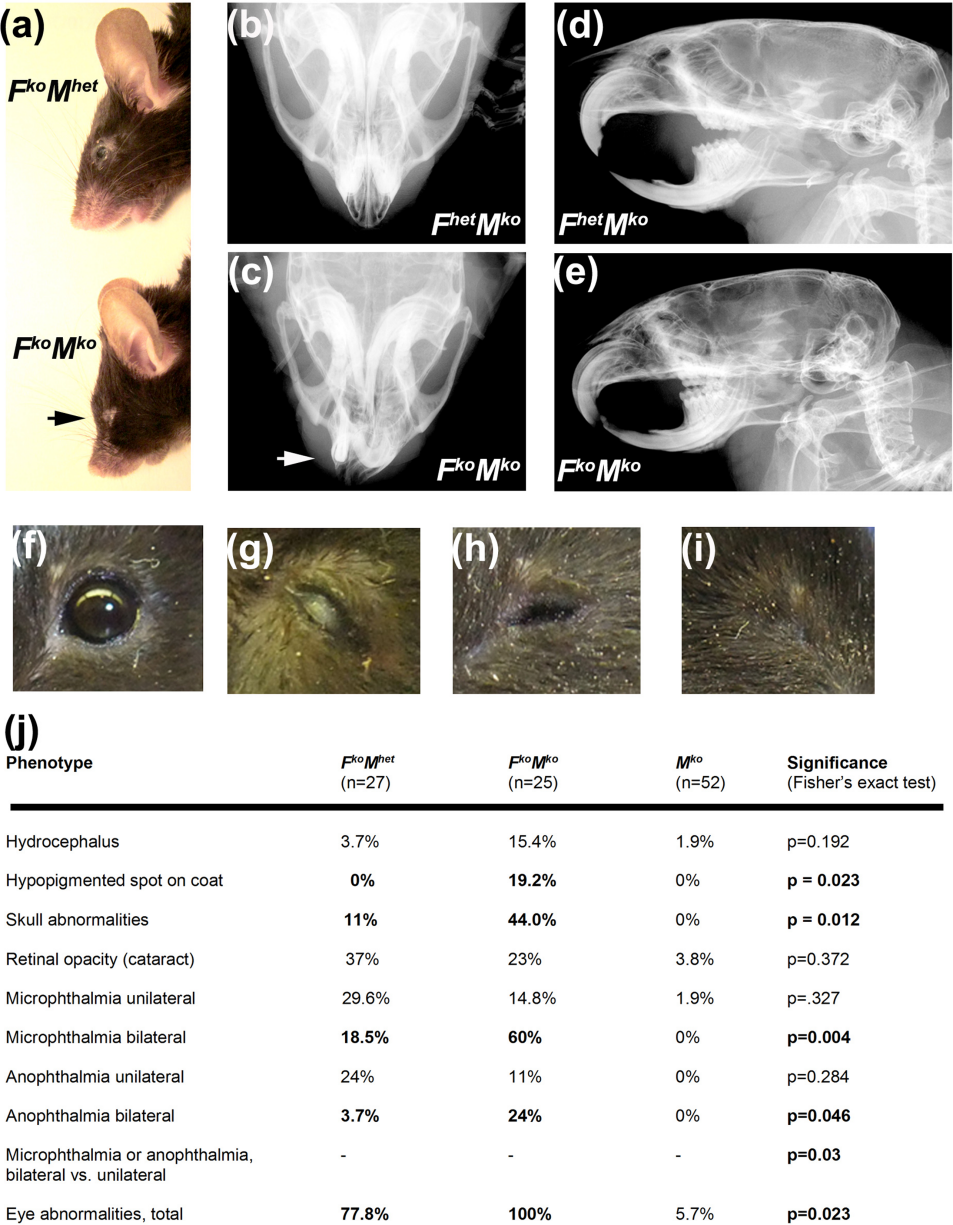
Increased prevalence of congenital defects in *F^ko^M^ko^* mice. (**a**) Representative image illustrating characteristic craniofacial features of *F^ko^M^ko^* mice compared to the normal appearance of *F^ko^M^het^* controls. (**b, c**) Representative X-ray of skulls showing deviated rostrum, micrognathia and abnormal dentition in *F^ko^M^ko^* mice (c) compared to that of *F^het^M^ko^* control (b). (**d, e**) Representative X-rays of skulls showing abnormal skull shape and pronounced microcephaly in *F^ko^M^ko^* mice (e) compared to that of *F^het^M^ko^* controls (d). (**f-i**). Representative images of diverse ocular defects observed. (f) Normal eye. (g) Corneal opacity/cataract. (h) Microphthalmia. (i) Anophthalmia. (**j**) Table indicating frequency of observed congenital abnormalities, *P*-values calculated by Fisher's exact test with significant differences in bold font.

In addition to the overall decrease in mass and size, *F^ko^M^ko^* mice exhibited dysmorphic facial features along with increased susceptibility to other congenital abnormalities (Figure 2). Typically, *F^ko^M^ko^* mice exhibited micrognathia and more pronounced foreheads than siblings (Figure 2A). In 11 of 26 *F^ko^M^ko^* mice examined (42.3%), X-ray analysis showed overt abnormalities in the overall shape and symmetry of the skull compared to three of 27 *F^ko^M^het^* mice (*P* = 0.0012; Figure 2B–E). All *F^ko^M^ko^* mice exhibited eye abnormalities (retinal opacity, microphthalmia and anophthalmia; Figure 2F–I) compared to *F^ko^M^het^* mice (77% incidence, *P* = 0.02), in particular the incidence of bilateral versus unilateral microphthalmia (*P* = 0.004) and bilateral versus unilateral anophthalmia (*P* = 0.046). Furthermore, a low percentage of *F^ko^M^ko^* mice showed additional abnormalities such as hypopigmentation of coat fur, and trended toward susceptibility to hydrocephalus (14.8% incidence) compared to *F^ko^M^het^* mice (3.7% incidence; Figure 2J). No differences in hematological parameters were observed among the genotypes examined (Supplementary Figure S1). Overall, *F^ko^M^ko^* mice exhibited increased severity and frequency of phenotypes observed in *F^ko^* mice as well as novel traits not observed in either *F^ko^* or *M^ko^* mice.

### FancC and Mus81 cooperate to ensure genome integrity *in utero*

**Figure 3.**
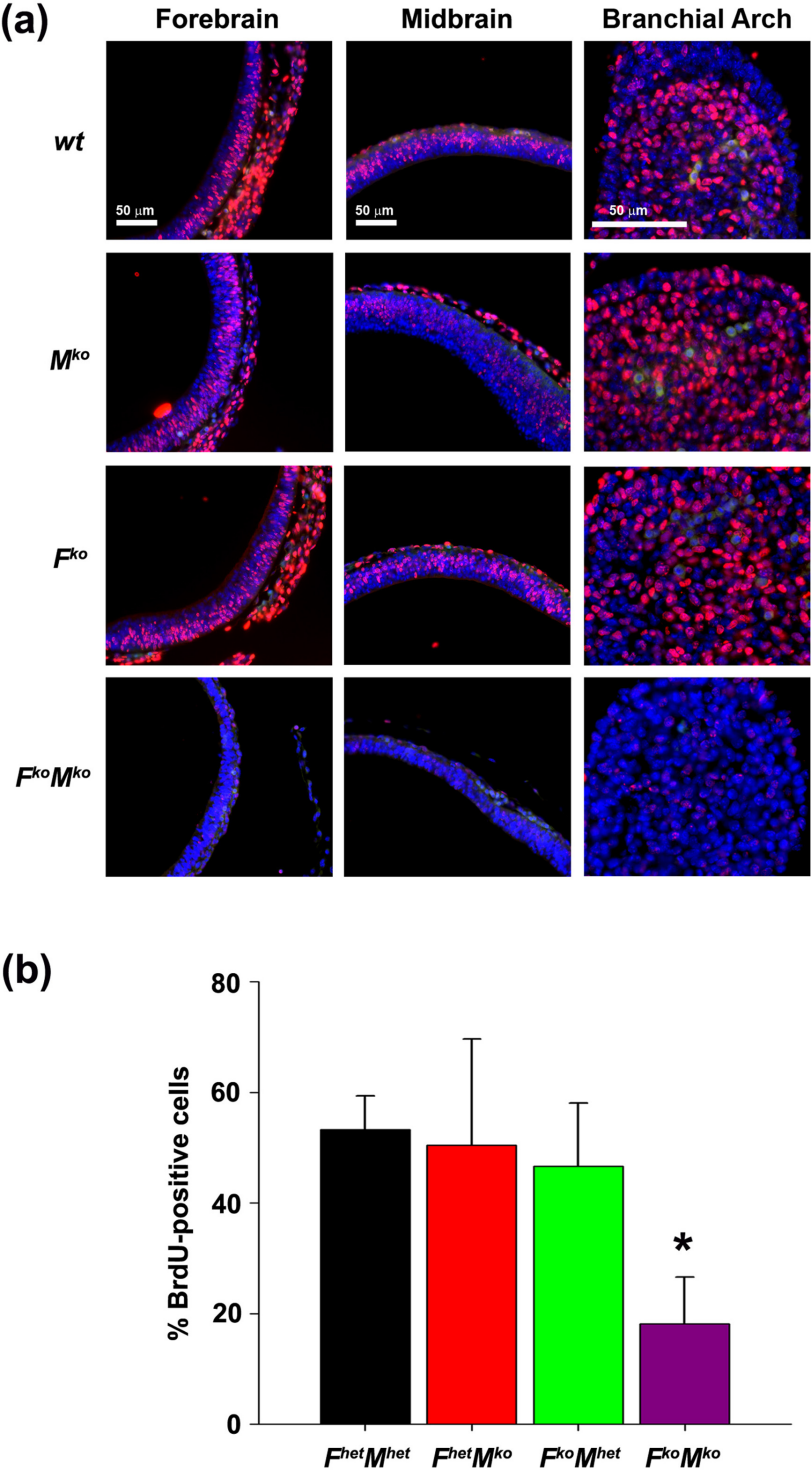
Proliferation defect in *F^ko^M^ko^* embryos. (**a**) BrdU incorporation (red) in E10.5 embryos; blue, DAPI (nuclei); yellow, erythrocyte auto fluorescence. (**b**) Quantification of % of BrdU-positive cells in branchial arch. Data represent the mean number of BrdU-labeled cells per genotype ±SD (*n* = 3). *P < 0.05 by one-way ANOVA followed by Holm–Sidak post hoc test for *F^ko^M^ko^* versus other genotypes.

**Figure 4.**
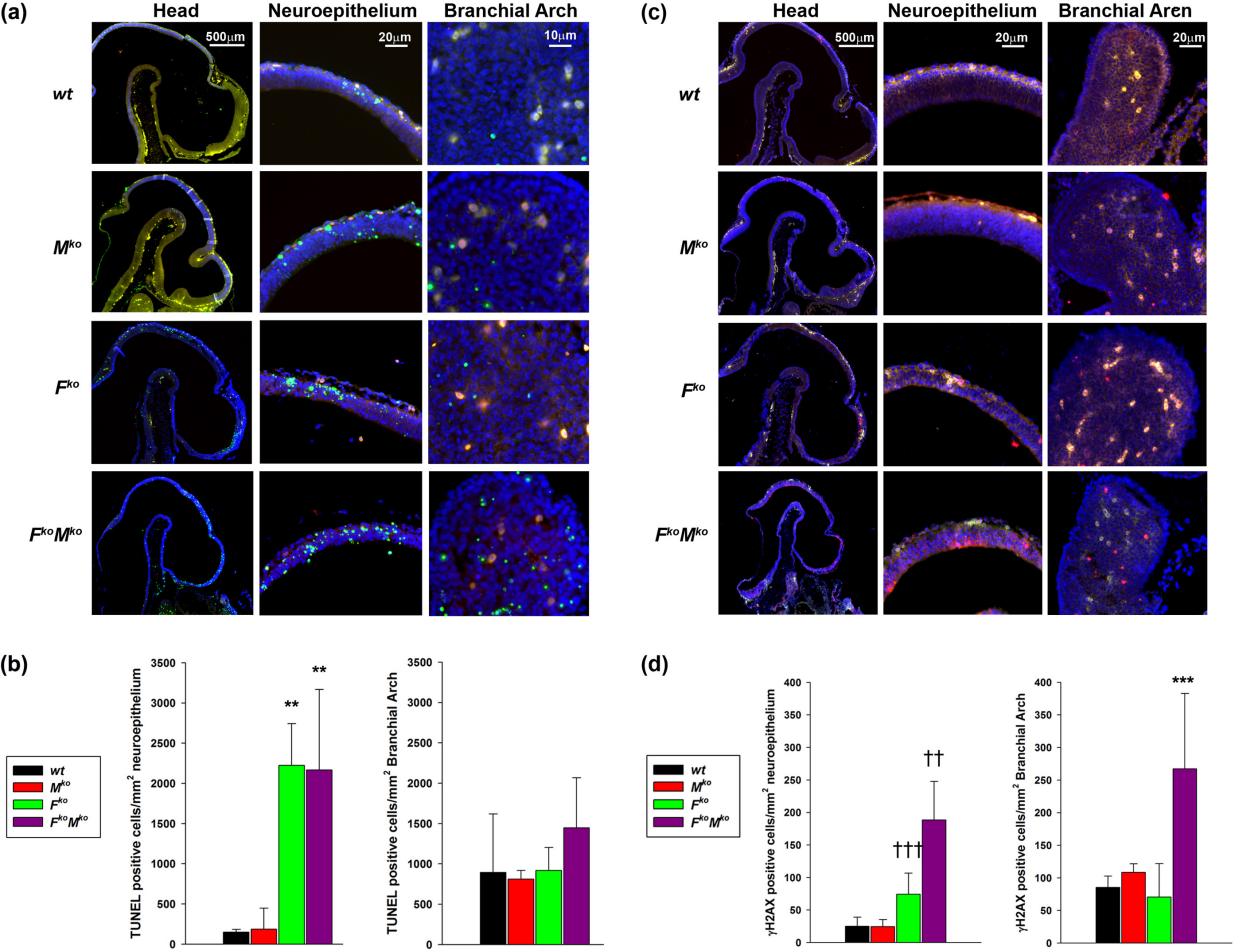
Propensity for apoptosis and DNA damage in *F^ko^M^ko^* embryos. (**a**) Representative TUNEL of head region, neuroepithelium and branchial arch from wild-type, *M^ko^*, *F^ko^*, and *F^ko^M^ko^* E10.5 embryos. (**b**) Quantification of TUNEL-positive cell/mm^2^ neuroepithelium and branchial arch. Data represent the mean number of TUNEL-positive cells per genotype ±SD (*n* = 3). **P < 0.01 for *F^ko^M^ko^* or *F^ko^* versus *wt* and *M^ko^* by one-way ANOVA followed by Holm–Sidak post hoc test. No differences in TUNEL-labeled cells were detected in the branchial arch. (**c**) Representative γH2AX staining in head, neuroepithelium and branchial arch from wild-type, *M^ko^*, *F^ko^* and *F^ko^M^ko^* E10.5 embryos. (**d**) Quantification of γH2AX-positive cells/mm^2^ in neuroepithelium and branchial arch. Data represent mean number of γH2AX-positive cells ±SD. †††*P* < 0.001 for *F^ko^M^ko^* versus *wt* and *M^ko^*, ††*P* < 0.01 for *F^ko^M^ko^* versus *wt* or *M^ko^*, ***P < 0.001 for *F^ko^M^ko^* versus all other genotypes assessed by one-way ANOVA followed by Holm–Sidak post hoc test.

In order to understand the underlying cause of the developmental delay *in utero*, we examined embryonic cell proliferation, apoptosis and DNA damage *in situ*. Compared to sibling controls, viable *F^ko^M^ko^* embryos at E10.5 and E11.5 showed a greatly reduced number of BrdU-positive cells, indicative of reduced proliferation (Figure 3A). In addition, the BrdU-positive cells that were present exhibited a lower level of BrdU incorporation per cell throughout the entire embryo (Figure 3A). When frequency of BrdU-positive cells in the branchial arch was quantified, *F^ko^M^ko^* embryos at E10.5 showed a significant decrease in the number of cells synthesizing DNA compared to sibling *F^het^M^het^* (*P* = 0.006), *F^het^M^ko^* (*P* = 0.012) and *F^ko^M^het^* (*P* = 0.018) embryos (Figure 3B). Compared to sibling controls, both *F^k^*^o^ and *F^ko^M^ko^* embryos at E10.5 showed a dramatic increase in the frequency of apoptotic cells specifically in the neuroepithelium of the developing embryonic brain (*P* < 0.01 versus *wild-type* and *M^ko^*; Figure 4A and B). Interestingly, the frequency of apoptotic cells was equivalent for all genotypes in the branchial arch, suggesting that loss of FancC renders embryos more susceptible to apoptosis in the forebrain and neuroepithelium, but this effect is specific for FancC loss only and is independent of Mus81 status. We examined the possibility that accumulated DNA damage might serve to trigger apoptosis or proliferation arrest in E10.5 embryos by comparing sibling embryos for the presence of γH2AX. Quantification of γH2AX-positive cells in both the neuroepithelium and branchial arch revealed that *F^ko^M^ko^* embryos exhibited a higher frequency of γH2AX-positive cells in both regions (*P* < 0.001 versus*wild-type* and *M^ko^*, *P* = 0.002 versus *F^ko^* for neuroepithelium, *P* < 0.001 versus all genotypes assessed for branchial arch; Figure 4C-D), suggesting that the accumulated DNA damage in the absence of both FancC and Mus81 triggered checkpoints leading to either apoptosis or proliferation arrest.

### FancC and Mus81 cooperate to ensure genome integrity *ex vivo*

**Figure 5.**
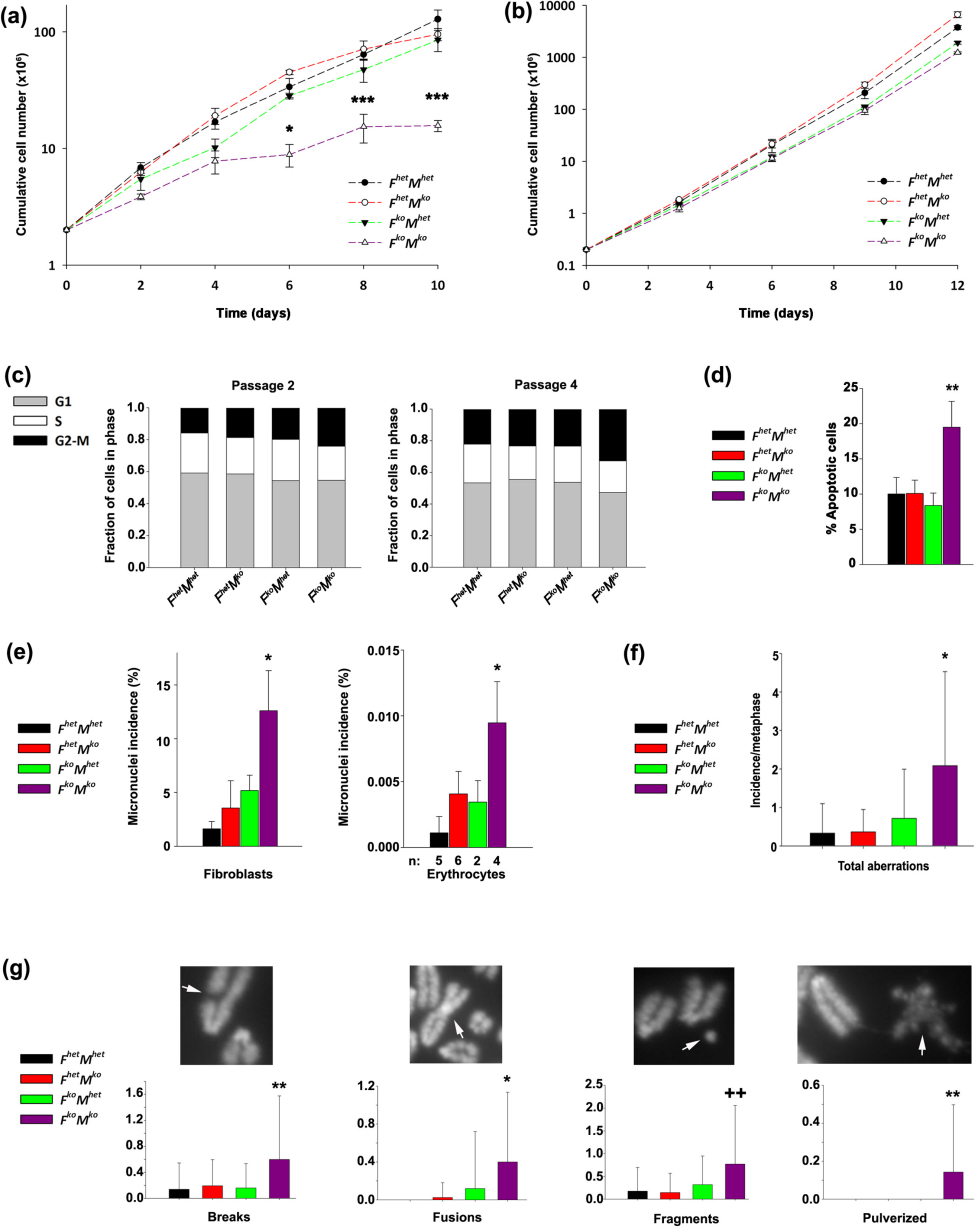
Compromised proliferation and chromosomal instability in *F^ko^M^ko^* primary fibroblasts. (**a**) Proliferation defect of *F^ko^M^ko^* primary fibroblasts. Data represent the mean of the cumulative cell number at each assessment day ±SD. *P < 0.05, ***P < 0.001 versus all genotypes by two-way ANOVA followed by Holm-Sidak posthoc test. (**b**) Proliferation of SV40-transformed fibroblasts. Data were analyzed by two-way ANOVA; however, no statistically significant differences were observed. (**c**) Passage-dependent accumulation of *F^ko^M^ko^* fibroblasts in G2/M. Significant differences in cell cycle were observed in *F^ko^M^ko^* cells at passage 4. Specifically, a significant accumulation of *F^ko^M^ko^* fibroblasts in G2/M was observed from passage 2 to passage 4. Data represent the average percentage of cells in each phase (*n* = 3) for a total of 100%. At passage 4, G1 phase, *P* < 0.001 versus all other genotypes. For the S phase, *P* < 0.05 versus all genotypes and for *F^ko^M^het^* versus *F^het^M^het^.* Data were analyzed by two-way ANOVA followed by Holm–Sidak post hoc test. (**d**) Increased propensity for *F^ko^M^ko^* fibroblasts to undergo spontaneous apoptosis. Data represent the mean percentage of cells in both early and late apoptosis (*n* = 3). **P < 0.01 versus all genotypes based on the results of one-way ANOVA followed by Holm–Sidak post hoc test. (**e**) Incidence of micronuclei in primary untreated embryonic fibroblasts and primary untreated erythrocytes,*P < 0.05 for *F^ko^M^ko^* versus all genotypes based on the results of one-way ANOVA followed by Holm–Sidak post hoc test; (**f**) frequency of total aberrations/metaphase in primary fibroblasts plotted as incidence/metaphase. Data represent the mean incidence per metaphase ±SD, *P < 0.05 versus all genotypes by one-way ANOVA followed by Holm–Sidak post hoc test. (**g**) Incidence of chromosome aberrations: breaks and pulverized **P < 0.01 versus all genotypes, fusions *P < 0.05 versus all genotypes, fragments ++*P* < 0.01 versus *F^het^M^ko^* and *F^het^M^het^*. All data were analyzed by one-way ANOVA followed by Holm–Sidak post hoc test.

Primary cultures of fibroblasts from *F^het^M^het^*, *F^het^M^ko^*, *F^ko^M^het^* and *F^ko^M^ko^* sibling embryos were propagated in order to establish if the observed defects in *F^ko^M^ko^* mice reflected a cell-intrinsic mechanism. Compared to fibroblasts from sibling controls, a pronounced proliferation defect was apparent in *F^ko^M^ko^* primary fibroblasts at each time of assessment (*P* between 0.05 and < 0.001 dependent upon passage; Figure 5A). Reversal of the growth defect was observed in *F^ko^M^ko^* fibroblasts following immortalization with SV40 (Figure 5B), suggesting that the observed loss of proliferation is at least partly due to p53-mediated checkpoint activation. Compared to *F^het^M^het^*, *F^het^M^ko^* and *F^ko^M^het^* cells*, F^ko^M^ko^* primary fibroblasts also showed a tendency to accumulate in the G2/M phases of the cell cycle, with increased arrest occurring in a passage-dependent manner (*P* < 0.001 for P4 G2/M, *P* < 0.05 for P4 S-phase; Figure 5C). Furthermore, *F^ko^M^ko^* primary fibroblasts showed a greater tendency to undergo passage-dependent apoptosis in culture (*P* < 0.01; Figure 5D). As *F^ko^M^ko^* embryos exhibited an increased incidence of γH2AX-positive cells, we examined whether elevated levels of spontaneous DNA damage were mirrored at the cellular level through quantification of micronuclei. We determined that primary *F^ko^M^ko^* fibroblasts exhibited an increased incidence of micronuclei compared to all controls (*P* < 0.05; Figure 5E, left panel). Strikingly, this susceptibility to accumulated DNA damage was also observed in the erythrocytes of *F^ko^M^ko^* adult mice (aged 6–8 months) enumerated for micronuclei (Howell-Jolly bodies), suggesting that the observed susceptibility to DNA damage was not specific to embryonic cells (*P* < 0.001 versus *F^het^M^het^*,*P* = 0.005 versus *F^het^M^ko^*,*P* = 0.030 versus *F^ko^M^het^*; Figure 5E, right panel).

The elevated micronuclei in *F^ko^M^ko^* cells may reflect an overall increase in total chromosomal instability and/or may be restricted to increases in specific aberrations. To query these possibilities, we conducted karyotype analysis of metaphases from untreated primary fibroblasts. In untreated primary fibroblasts, total aberrations per metaphase were highest in *F^ko^M^ko^* cells (Figure 5F). In primary cells, the incidence of breaks (*P* = 0.001 versus *F^het^M^het^*,*P* = 0.009 versus *F^het^M^ko^* or *F^ko^M^het^*) and fusions (*P* < 0.001 versus*F^het^M^het^*,*P* = 0.001 versus*F^het^M^ko^*,*P* = 0.036 versus*F^ko^M^het^*) was significantly higher than in cells of other genotypes. Significant increases in the prevalence of fragments were observed in *F^ko^M^ko^* cells relative to *F^het^M^het^* and *F^het^M^ko^* cells (*P* = 0.002), but not versus *F^ko^M^het^* cells (Figure 5G). Of interest, novel chromosomal aberrations that resemble pulverized chromosomes were identified only in metaphases from primary *F^ko^M^ko^* cells and were not detected in cells of other genotypes (*P* < 0.001 versus*F^het^M^het^* and*F^het^M^ko^*,*P* = 0.002 versus*F^ko^M^het^*). These novel structures are thought to be produced as a result of catastrophic mitotic events (34) and exhibit a disordered structure with large regions of decondensed chromatin, evident from the low intensity of DAPI staining (Figure 5G). Taken together, the cooperative effect of FancC and Mus81 in maintenance of chromosome integrity in fibroblasts mirrors the *in utero* requirement for both pathways.

### FancC and Mus81 cooperate in repair of crosslink damage but are epistatic in the response to replicative stress

**Figure 6.**
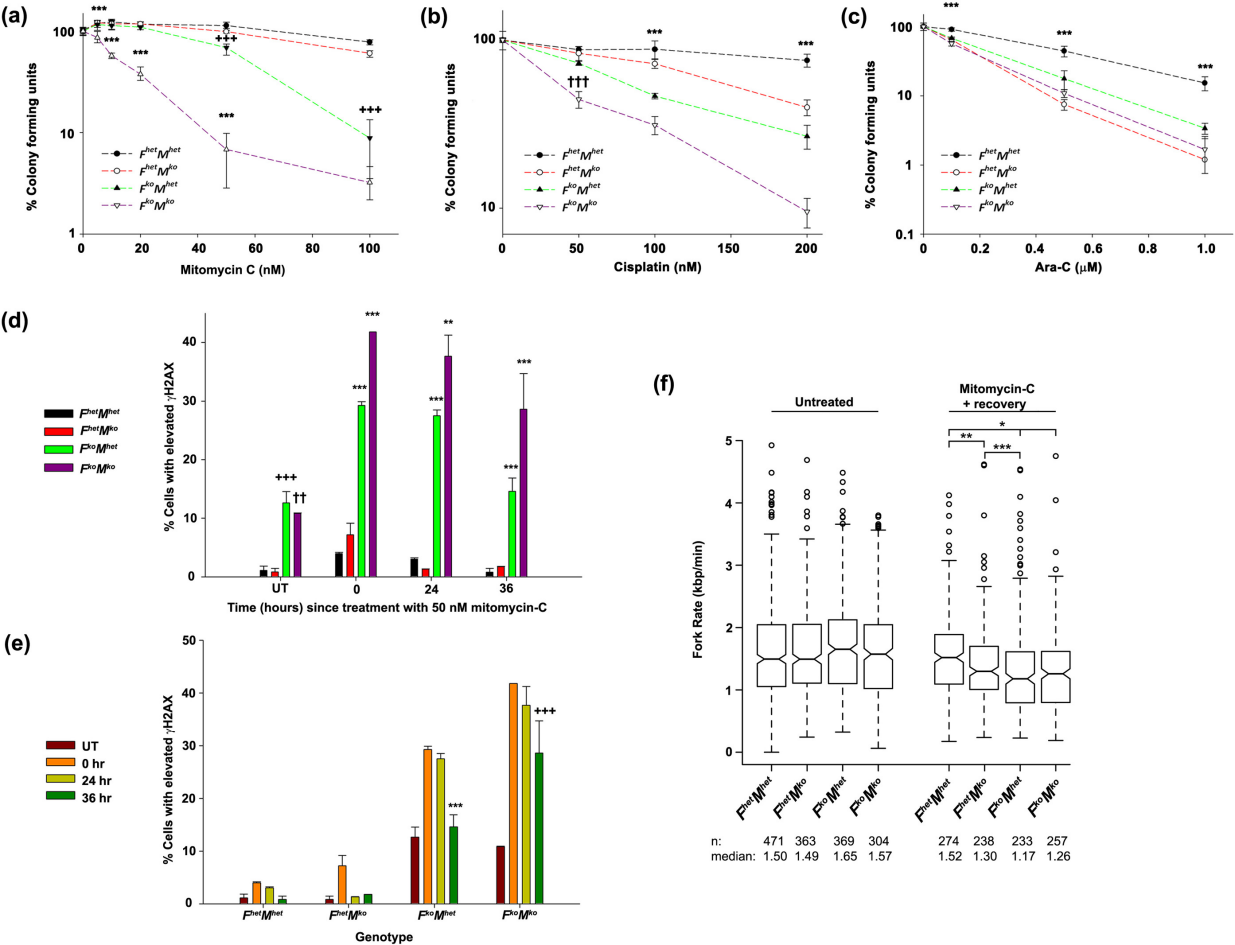
Response of *F^ko^M^ko^* cells to DNA damaging agents. (**a-c**) Sensitivity of immortalized fibroblasts to mitomycin-C (a), cisplatin (b) and Ara-C (c) by clonogenic assay. Data represent mean percentage of colony forming units compared to control (untreated *F^het^M^het^*) per dose assessed ±SD. For (a), ***P < 0.001 for *F^ko^M^ko^* versus all genotypes, ±±*P* < 0.01 for *F^het^M^ko^* versus *F^het^M^het^*, +++*P* < 0.001 for *F^ko^M^het^* or *F^ko^M^ko^* versus *F^het^M^ko^* and *F^het^M^het^*. For (b), ***P < 0.001 for all genotypes versus each other, †††*P* < 0.001 for *F^ko^M^ko^* versus all genotypes. For (c), ***P < 0.001 for *F^het^M^het^* versus all genotypes. All data were analyzed by two-way ANOVA followed by Bonferroni post hoc test. (**d, e**) Percentage of primary fibroblasts with elevated γH2AX 0, 24 or 36 h after mitomycin-C exposure for 24 h (UT, untreated) with data grouped according to time point (d) or genotype (e). Data represent mean % cells ± SD with elevated γH2AX (*n* = 3) assessed by two-way ANOVA followed by Bonferroni posthoc test. For (d), ***P < 0.001 for *F^ko^M^ko^* versus all genotypes or *F^ko^M^het^* versus *F^het^M^het^* or *F^het^M^ko^*, **P < 0.01 for *F^ko^M^ko^* versus all genotypes, +++*P* < 0.001 for *F^ko^M^het^* versus *F^het^M^het^* and *F^het^M^ko^*, ††*P* < 0.01 for *F^ko^M^ko^* versus *F^het^M^het^* or *F^het^M^ko^*. For (e), ***P < 0.001 for 36 h versus 24 h and 0 h but not UT, +++*P* < 0.005 for 36 h versus 24 h, 0 h and UT. (**f**) Replication fork rate in primary fibroblasts. Whiskers represent 1.5 × interquartile range. *P < 0.001 for *F^het^M^het^* versus *F^ko^M^het^* or *F^ko^M^ko^*, **P = 0.04 for *F^het^M^het^* versus *F^het^M^ko^*, ***P = 0.018 for *F^het^M^ko^* versus *F^ko^M^het^*. Data were assessed using Mann–Whitney U-test for non-parametric data.

To ascertain sensitivity to DNA crosslink damage, clonogenic assays with immortalized fibroblasts of each genotype were conducted in the presence of mitomycin-C or cisplatin. As expected, *F^het^M^ko^* and *F^ko^M^het^* cells were hypersensitive to mitomycin-C compared to *F^het^M^het^*, with *F^ko^M^het^* cells showing a greater sensitivity to this agent at the 50–100-nM doses (*P* < 0.001) than *F^het^M^ko^* cells (Figure 6A). *F^ko^M^ko^* cells were remarkably more sensitive than cells from all other genotypes at 5–50-nM doses (*P* < 0.001). A similar ranking of sensitivity by genotype was observed when cells were exposed to cisplatin (*F^ko^M^ko^* > *F^ko^M^het^* > *F^het^M^ko^* > *F^het^M^het^*) with *F^ko^M^ko^* cells more sensitive than cells from all other genotypes at all doses (*P* < 0.001; Figure 6B). To determine if this sensitivity ranking was specific for crosslink damage or also included agents that generate replication-fork associated DNA damage, cells of all genotypes were exposed to Ara-C. At all doses tested, *F^ko^M^ko^*, *F^ko^M^het^* and *F^het^M^ko^* cells were significantly more sensitive than *F^het^M^het^* cells (*P* < 0.001; Figure 6C). In contrast to results with crosslinking agents, *F^ko^M^ko^* cells showed equivalent sensitivity to cells deficient in either Mus81 or FancC. Our findings indicate FA signaling and Mus81 operate in parallel pathways with respect to crosslink resistance, but in the same pathway with respect to replication fork-associated DNA damage.

To explore whether sensitivity to crosslink damage in *F^ko^M^ko^* cells reflected deficiencies in DNA repair, we scored incidence of immortalized fibroblasts with elevated γH2AX as a function of time following removal of cells from 50-nM mitomycin-C for 24 h (Figure 6D and E). *F^ko^M^ko^* cells exhibited the highest percentage of cells with elevated γH2AX at 0 (*P* < 0.001 versus all genotypes), 24 (*P* < 0.001 versus *F^het^M^het^* and *F^het^M^ko^*,*P* = 0.002 versus *F^ko^M^het^*) and 36 h (*P* < 0.001 versus all genotypes) following removal of mitomycin-C, whereas *F^ko^M^het^* cells showed the next highest percentages at the same time points (*P* < 0.001 at all time points compared to *F^het^M^het^* and *F^het^M^ko^*). *F^ko^M^het^* cells showed elevated γH2AX at 0 h and 24 h compared to untreated (*P* < 0.001); however, by 36 h, γH2AX levels were equivalent to untreated (*P* = 1; Figure 6E). In contrast, *F^ko^M^ko^* cells showed elevated levels at all time points compared to untreated (*P* < 0.005), indicative of a greater deficiency in DNA repair compared to *F^ko^M^het^* cells. To determine if these differences in repair rate could be attributed to an intrinsic defect in maintenance of DNA replication forks, fork velocity was measured in both untreated fibroblasts and fibroblasts allowed to recover for 6 h following exposure to 50-nM mitomycin-C for 24 h (Figure 6F). Untreated fibroblasts of all four genotypes showed equivalent replication fork velocity. After 6-h recovery following mitomycin-C exposure, fork velocity remained lower in *F^ko^M^het^* (*P* < 0.001), *F^ko^M^ko^* (*P* < 0.001) and in *F^het^M^ko^* (*P* = 0.04) cells compared to *F^het^M^het^* cells, however fork rate in *F^ko^M^ko^* was not significantly lower than *F^het^M^ko^* or *F^het^M^ko^*. Taken together, these findings suggest that recovery of replication fork velocity following mitomycin-C exposure requires FancC and Mus81, but that the exacerbated repair defect in *F^ko^M^ko^* cells cannot be attributed to impaired recovery of replication fork velocity.

### Distinct roles for FancC and Mus81 in the repair of chromosomal lesions

**Figure 7.**
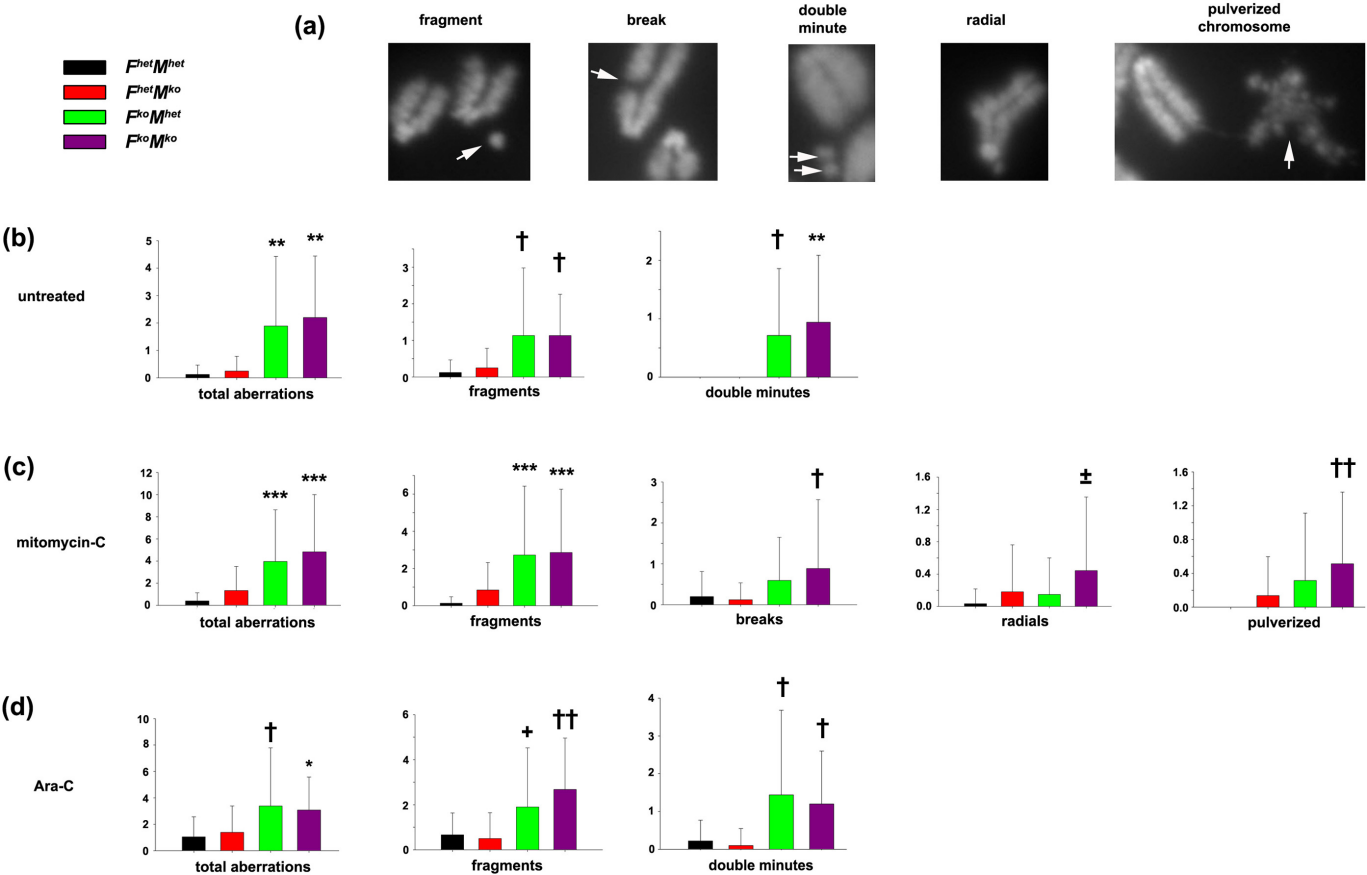
Genomic instability in *F^ko^M^ko^* immortalized cells treated with DNA damaging agents. (**a**) Representative chromosomal aberrations scored (arrows). (**b**) Untreated immortalized fibroblasts: total aberrations, fragments and double minutes. Data represent the mean incidence per metaphase ±SD. **P < 0.005 for *F^ko^M^het^* or *F^ko^M^ko^* versus *F^het^M^het^* or *F^het^M^ko^*, †*P* < 0.05 for *F^ko^M^ko^* or *F^ko^M^het^* versus *F^het^M^ko^* or *F^het^M^het^.* (**c**) Immortalized fibroblasts exposed to mitomycin-C: total aberrations, fragments, breaks, radials and pulverized chromosomes. ***P < 0.001 for *F^ko^M^ko^* or *F^ko^M^het^* versus *F^het^M^het^* and *F^het^M^ko^*, †*P* < 0.05 for *F^ko^M^ko^* versus *F^het^M^het^* or *F^het^M^ko^*, ±*P* < 0.05 for *F^ko^M^ko^* versus *F^het^M^het^*, ††*P* < 0.01 for *F^ko^M^ko^* versus *F^het^M^het^* or *F^het^M^ko^*. (**d**) Immortalized fibroblasts exposed to Ara-C: total aberrations, fragments and double minutes. *P < 0.05 for *F^ko^M^ko^* versus *F^het^M^het^*, †*P* < 0.05 for *F^ko^M^het^* or *F^ko^M^ko^* versus *F^het^M^het^* or *F^het^M^ko^*, +*P* < 0.05 for *F^ko^M^het^* versus *F^het^M^ko^*, ††*P* < 0.005 for *F^ko^M^ko^* versus *F^het^M^ko^* and *F^het^M^het^*. All data analyzed by one-way ANOVA followed by Holm–Sidak post hoc.

To determine whether the heightened repair defect in *F^ko^M^ko^* cells reflects distinct contributions by FancC and Mus81 in the repair of specific lesions, we analyzed metaphase chromosomes from immortalized fibroblasts that were either untreated or exposed to either mitomycin-C or Ara-C (Figure 7). *F^ko^M^ko^*-immortalized cells demonstrated a significantly higher number of chromosomal aberrations per metaphase than either *F^het^M^het^* (*P* < 0.001) or *F^het^M^ko^* (*P* = 0.001) cells, however they did not differ significantly from *F^ko^M^het^* cells with regard to total aberrations. *F^ko^M^het^-*immortalized fibroblasts demonstrated a significantly higher number of aberrations per metaphase than *F^het^M^het^* or *F^het^M^ko^* cells (*P* = 0.003; Figure 7B). Interestingly, pulverized chromosomes were not detected in untreated immortalized fibroblasts. Instead, significant changes in the incidence of aberrations were restricted to fragments and another distinct aberration appearing as paired chromosomal fragments or ‘double minutes’. The incidence of fragments was higher in both *F^ko^M^ko^* and *F^ko^M^het^* cells (*P* = 0.03 versus *F^het^M^ko^* and *P* = 0.02 versus *F^het^M^het^*), similarly incidence of double minutes was higher in *F^ko^M^ko^* (*P* < 0.001 versus *F^het^M^ko^* and *P* = 0.001 versus *F^het^M^het^*) and *F^ko^M^het^* cells (*P* = 0.009 versus *F^het^M^ko^* and *P* = 0.01 versus *F^het^M^het^*).

The spectrum of induced chromosomal aberrations in immortalized cells exposed to mitomycin-C was markedly different compared to untreated cells or cells exposed to Ara-C (Figure 7C and D). In mitomycin-C-treated cells, elevated incidence of fragments was observed for both *F^ko^M^het^* and *F^ko^M^ko^* cells (*P* < 0.001 versus *F^het^M^het^* and *F^het^M^ko^* but not versus each other), whereas *F^ko^M^ko^* cells showed a higher propensity for breaks (*P* < 0.001 versus *F^het^M^ko^*, *P* = 0.03 versus *F^het^M^het^*), radials (*P* = 0.025 versus *F^het^M^het^*) and pulverized chromosomes (*P* = 0.003 versus *F^het^M^het^*, *P* = 0.006 versus *F^het^M^ko^*; Figure 7C). Incidentally, radials and pulverized chromosomes were only observed in immortalized cells when exposed to mitomycin-C and pulverized chromosomes were never observed in immortalized *F^het^M^het^* cells exposed to this agent. Ara-C treatment did not markedly alter the spectrum of particular aberrations compared to untreated cells in that the incidence of fragments was greater in *F^ko^M^ko^* cells (*P* = 0.001 versus *F^het^M^het^*,*P* < 0.001 versus *F^het^M^ko^*) and in *F^ko^M^het^* cells (*P* = 0.037 versus *F^het^M^ko^*), whereas double minutes were greater in *F^ko^M^het^* cells (*P* = 0.011 versus *F^het^M^het^*;*P* = 0.003 versus *F^het^M^ko^*) and in *F^ko^M^ko^* cells (*P* = 0.03 versus *F^het^M^het^*,*P* = 0.012 versus *F^het^M^ko^*) exposed to this agent (Figure 7D). Taken together, these findings indicate that FancC and Mus81 have distinct roles in ensuring the repair of agent-specific lesions.

## DISCUSSION

Recent advances in understanding how the FA pathway operates at a molecular level contrast with our poor understanding of how disruptions in this pathway lead to human FA disease traits. Although hematological defects exhibited by FA patients can be recapitulated by co-deletion of murine Aldh2 and *FancD2* (35), the observed phenotypes of many mouse models of FA do not accurately mimic the human traits of this disease, yet these mouse models exhibit the cellular sensitivity to crosslinking agents observed in cells from human patients (16). The initial aim of our study was to establish if FancC and Mus81 belong in distinct or parallel pathways, or if Mus81 is actually a member of the FA pathway. We found that Mus81 operates outside of the FA pathway with respect to ICL repair and that, interestingly, mice deficient in both pathways exhibit phenotypes that more closely resemble traits observed in many human FA patients.

*F^ko^* mice were susceptible to perinatal or embryonic lethality of unknown etiology previously described in several other FA mouse mutants (10,30,36–46). In contrast, Mus81 deficiency alone does not impair fetal viability, suggesting that Mus81 plays a minor *in utero* role in the repair of lesions compared with the FA pathway. Surprisingly, concomitant inactivation of both Mus81 and FancC was sufficient to significantly increase embryonic lethality compared to *F^ko^* mice alone and increase the incidence of congenital abnormalities observed. There was a dramatic decrease in viability of *F^ko^M^ko^* embryos from E9.5 to E12.5 compared to *F^ko^* embryos, indicating that Mus81 plays a critical backup role for the FA pathway during this period of development. Previously, embryonic exposure to mitomycin-C and Ara-C between ∼E8.5 and E12.5 has been shown to result in several of the congenital malformations we observed in *F^ko^M^ko^* mice, including small size, craniofacial malformations and eye defects (47,48). As the FA pathway is crucial to the repair of damage caused by both mitomycin-C and Ara-C, we infer that this particular stage of embryonic development is extremely sensitive to endogenous damage, leading to congenital malformations. Interestingly, loss of either FancC or Mus81 can result in distinct cell-specific outcomes. FancC deficiency is sufficient to induce increased apoptosis in the neuroepithelium, consistent with previous findings (49), whereas loss of both FancC and Mus81 triggers reduced proliferation. Accordingly, the increased apoptosis and reduced proliferation in the neuroepithelium and other regions of *F^ko^M^ko^* embryos may contribute to the increased severity of observed defects in *F^ko^M^ko^* mice that survive to birth. Although *F^ko^M^ko^* mice do not show overt pancytopenia, they exhibit an increased incidence of other clinical traits associated with human FA. Short stature due to impaired postnatal growth is a common feature of individuals with FA (50,51). Furthermore, the microcephaly, ocular deformities, frontonasal dysplasia and mandibular micrognathism in *F^ko^M^ko^* mice phenocopy disease traits in some FA individuals (52–54). Taken together, our findings suggest that failure to maintain a basal level of repair capacity contributes to the increased incidence of congenital abnormalities.

Following characterization of the *F^ko^M^ko^* mice on a pre- and post-natal level, we turned our attention to the molecular characterization of this model using an *ex vivo* approach. Primary *F^ko^M^ko^* embryonic fibroblasts exhibit a progressive impediment in proliferation, cell cycle arrest and increased incidence of apoptosis compared to fibroblasts deficient in either *F^ko^* or *M^ko^* alone. This proliferative response is attenuated in SV40-transformed cells, suggesting impaired p53-induced checkpoint responses. Given that FA and Mus81 cooperate in parallel pathways to ensure proper development *in utero*, it was of interest to investigate possible interactions in response to damage by ICLs. With respect to sensitivity to mitomycin-C and cisplatin, concomitant loss of Mus81 and the FA pathway led to greater sensitivity to these agents compared to loss of either pathway alone. Our findings are consistent with previous reports that ICL-induced monoubiquitination of FANCD2 is normal in Mus81-deficient cells (26). Recovery from DNA damage following exposure to mitomcycin-C was impaired in *F^ko^M^ko^* cells, as γH2AX levels in *F^ko^M^ko^* cells remained significantly elevated throughout the recovery time period, while γH2AX signal in *F^ko^* cells falls to basal levels after the recovery period. Differences in the rate of disappearance of γH2AX indicate that *F^ko^M^ko^* cells are defective in repairing ICL damage and also indicate a significant role for Mus81 in the removal of crosslinks in the absence of FA. The role of Mus81, however, appears to be minor compared to the FA pathway given that Mus81 deficient cells do not see a significant increase in γH2AX signal with ICL damage. Although the FA pathway (55–59) and Mus81 (27,60–62) both function in replication fork restart following DNA damage, the observed sensitization to mitomycin-C in *F^ko^M^ko^* cells is unlikely to be due to combined action by these two pathways on replication fork dynamics. The two pathways operate epistatically in the repair of compromised replication forks in that *F^ko^M^ko^* cells treated with the replicative stress-inducing agent Ara-C were no more sensitive to this agent than either *F^ko^* or *M^ko^* cells alone. With respect to agents that cause replicative stress, it has been suggested that Mus81 facilitates restart of DNA synthesis through its ability to generate double-strand breaks as a backup response under conditions of chronic stress, when other pathways have been exhausted (27,63). Our findings suggest that FA and Mus81 operate in the same pathway in response to replicative stress, but in parallel pathways with respect to crosslink repair.

Among FA patients, the single uniting feature is the sensitivity of patient cells to clastogenic chromosomal damage via crosslinking agents. Based on our findings regarding proliferation and ICL sensitivity in *F^ko^M^ko^* cells, we sought to examine if combined loss of these pathways results in greater chromosomal instability. Fibroblasts and erythrocytes deficient in both the FA pathway and Mus81 exhibited higher levels of micronuclei, which are often signs of chromosomal breakage or lagging chromosomes following mitotic dysfunction (64–67). Accordingly, we examined the incidence and type of chromosomal aberrations that occur in *F^ko^M^ko^* cells. Surprisingly, karyotype analysis revealed that loss of either FA or Mus81 pathways, or both simultaneously, results in distinct outcomes with respect to the appearance of chromosomal abnormalities. In primary fibroblasts, *F^ko^M^ko^* cells showed the highest incidence of aberrations normally associated with chromosomal instability, including fragments, breaks and fusions. Of great interest was the observation of novel chromosomal arrangements that were only detected in primary cells and in immortalized cells treated with mitomycin-C. ‘Pulverized chromosomes’ exhibited a disordered structure and largely appeared to be composed of decondensed DNA. Recent studies have observed chromosome decondensation in cells depleted of Bloom Syndrome helicase and structure-specific nucleases that compromise the ability to resolve HJs (11,12,68). It is tempting to speculate that the pulverized chromosomes we have observed in unchallenged *F^ko^M^ko^* primary fibroblasts arise from a similar process. HJ processing is required during cell division to prevent sister chromatid entanglements or unresolved replication structures that might interfere with normal chromosome condensation and therefore prevent the generation of segregation defects and chromosome-shattering events (chromothripsis) during mitosis (34,68–71). In contrast to primary cells, small bridged acentric fragments that have the general appearance of ‘double minutes’ were consistently observed in immortalized *F^ko^M^ko^* and *F^ko^* cells compared to *M^ko^* and control cells. Interestingly, these double minute structures were present following exposure to Ara-C, whereas mitomycin-C exposure increased the overall incidence of pulverized chromosomes and radials, but suppressed the appearance of double minutes. Overall, our karyotypic analysis suggests that FancC and Mus81 reside in parallel pathways that safeguard chromosomal integrity and that untreated immortalized cells show a greater dependence on the FA pathway compared to the Mus81 pathway in the maintenance of genomic stability. Following mitomycin-C exposure, the impact of Mus81 deficiency becomes more apparent in the karyotypes from *F^ko^M^ko^* metaphases. The need for one pathway over the other appears to depend on the spectrum and incidence of induced DNA lesions.

The appearance and incidence of distinct karyotypic lesions for each genotype and treatment likely represent distinct contributions or requirements for the FA pathway and/or Mus81 in resolving HJs or related intermediates that arise during DNA repair. Recent studies indicate that sister chromatid exchanges associated with Bloom Syndrome helicase deficiency may utilize a distinct repertoire of nucleases compared to those associated with mitomycin C-induced damage, and that the role of these structure-specific nucleases in HJ resolution is independent of other repair processes in response to ICLs or replicative stress (10–13). It is known that ICLs generate sister chromatid exchanges (SCEs) during DNA replication. In human cells, these SCEs were found to be dependent on SLX1 and MUS81 (11,12). However, in murine cells it appears that the essential targets of SLX1-SLX4 and MUS81 in ICL repair are structures other than HJs (10).

We have shown that the FA pathway and Mus81 act in parallel not only in the resolution of ICL damage but also in mammalian developmental processes. Our findings imply that loss of both pathways contributes to accumulation of DNA damage past a critical threshold that permits normal development. Although increased susceptibility to crosslink damage appears to render mice more susceptible to increased prevalence and severity of congenital malformations typically seen in FA patients, the possibility of a similar relationship in humans remains to be identified. To date, the exact cause and variable penetrance for the array of symptoms associated with FA disease have yet to be determined. The *F^ko^M^ko^* mouse model affords new opportunities to track *in utero* and *in vivo* consequences of FA disruption during early embryogenesis.

## SUPPLEMENTARY DATA

Supplementary Data are available at NAR Online.

SUPPLEMENTARY DATA
