# Supplementary material for: Fanconi anemia signaling and Mus81 cooperate to safeguard development and crosslink repair
Source: Nucleic Acids Res. 2014 Jul 23;42(15):9807–20. doi: 10.1093/nar/gku676 (PMC4150781; doi:10.1093/nar/gku676)
Supplement: SUPPLEMENTARY DATA [file supp_42_15_9807__index.html]

Fanconi anemia signaling and Mus81 cooperate to safeguard development and crosslink repair — Fanconi anemia signaling and Mus81 cooperate to safeguard development and crosslink repair — SUPPLEMENTARY DATA 

# Fanconi anemia signaling and Mus81 cooperate to safeguard development and crosslink repair

## SUPPLEMENTARY DATA

**Files in this Data Supplement:**

- SUPPLEMENTARY DATA
