# Supplementary material for: Fanconi anemia signaling and Mus81 cooperate to safeguard development and crosslink repair
Source: Nucleic Acids Res. 2014 Jul 23;42(15):9807–20. doi: 10.1093/nar/gku676 (PMC4150781; doi:10.1093/nar/gku676)

Supplementary Table S1. $F^{ko}M^{ko}$ and F^{ko} mice are born at submendelian ratios. Viability of $F^{ko}M^{ko}$ and F^{ko} mice was assessed by scoring viable progeny of intercrosses at different stages of development. Each column represents the total number of embryos/pups observed with the indicated genotype. Percentage values in brackets represent the number of viable embryos/pups of a given genotype divided by total number of progeny obtained. p- values were calculated by χ^2 test.

Father	Mother	time	total #	F^{ko} expected #	F^{ko} observed #	$F^{ko}M^{ko}$ expected #	$F^{ko}M^{ko}$ observed #	*P* value
F^{het}	F^{het}	Birth	215	54 (25%)	33 (15%)			<0.001
$F^{het}M^{het}$	$F^{het}M^{ko}$	Birth	270			26 (12.5%)	9 (3%)	<0.005
$F^{het}M^{ko}$	$F^{het}M^{ko}$	Birth	245			61 (25%)	25 (10%)	<0.005
F^{het}	F^{het}	E9.5	48	12	8 (17%)			
		E10.5	71	18	15 (21%)			
		E11.5	17	4	3 (18%)			
		E12.5	32	8	5 (16%)			
$F^{het}M^{ko}$	$F^{het}M^{ko}$	E9.5	57			14	11 (19%)	
		E10.5	56			14	16 (29%)	
		E11.5	37			9	5 (14%)	
		E12.5	41			10	4 (10%)	

Supplementary Table S2. Percentage of embryos with observed abnormalities.

	Genotype	Total Embryos	Overall Small	Overall Delay	Head Small	Head Delay	Eye Defects	Total Anomalies
**E9.5**	*Wild type*	9	11.11	0.00	0.00	22.22	33.33	44.44
	F^{ko}	4	50.00	0.00	0.00	0.00	0.00	50.00
	M^{ko}	10	10.00	0.00	0.00	0.00	0.00	10.00
	$F^{ko}M^{ko}$	11	27.27	27.27	9.09	27.27	63.64	90.91
**E10.5**	*Wild type*	13	15.38	0.00	7.69	7.69	7.69	15.38
	F^{ko}	15	33.33	0.00	6.67	0.00	26.67	46.67
	M^{ko}	18	5.56	5.56	0.00	5.56	5.56	11.11
	$F^{ko}M^{ko}$	16	43.75	25.00	18.75	43.75	6.25	75.00
**E11.5**	*Wild type*	7	14.29	14.29	0.00	14.29	14.29	28.57
	F^{ko}	3	33.33	33.33	33.33	33.33	66.67	66.67
	M^{ko}	10	10.00	0.00	0.00	0.00	0.00	10.00
	$F^{ko}M^{ko}$	5	80.00	0.00	60.00	20.00	40.00	100.00
**E12.5**	*Wild type*	8	0.00	0.00	0.00	0.00	0.00	0.00
	F^{ko}	5	20.00	0.00	0.00	0.00	80.00	80.00
	M^{ko}	8	0.00	0.00	0.00	0.00	0.00	0.00
	$F^{ko}M^{ko}$	4	25.00	0.00	50.00	0.00	50.00	50.00

Supplementary Figure S1. Hematological analysis. (**a**) Peripheral erythrocyte concentration. (**b**) Peripheral white cell concentration. (**c**) Peripheral neutrophil concentration. (**d**) Peripheral platelet concentration.

Supplementary Figure S1

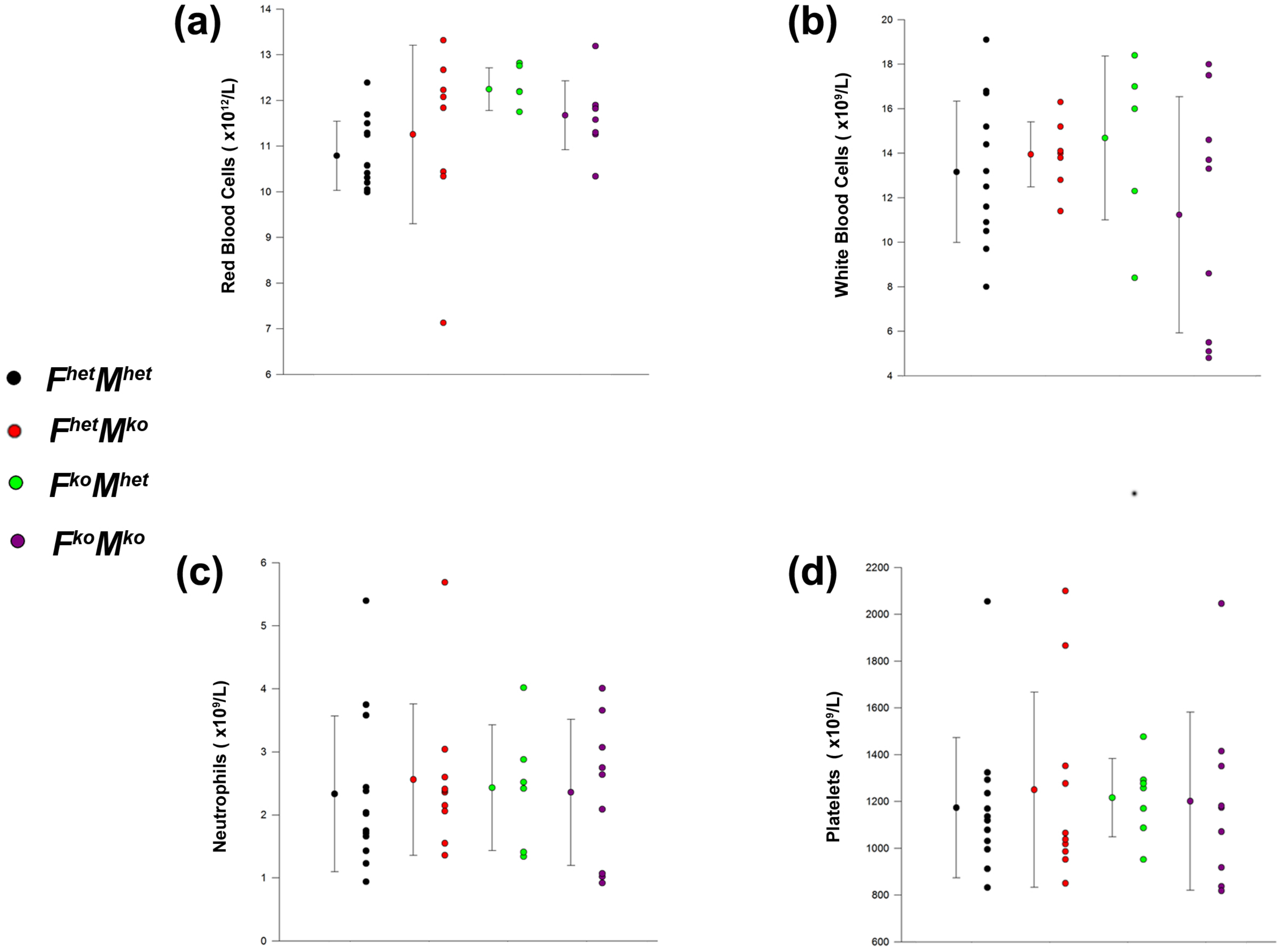

Supplement: SUPPLEMENTARY DATA [file supp_gku676_nar-01525-d-2014-File009.pdf]
